# Gender Differences in Psychosocial Outcomes and Coping Strategies of Patients with Colorectal Cancer: A Systematic Review

**DOI:** 10.3390/healthcare11182591

**Published:** 2023-09-20

**Authors:** Junrui Zhou, Zhiming Wang, Xuan Chen, Qiuping Li

**Affiliations:** Wuxi School of Medicine, Jiangnan University, Wuxi 214122, China; 6212807054@stu.jiangnan.edu.cn (J.Z.); 6212807040@stu.jiangnan.edu.cn (Z.W.); 6212807006@stu.jiangnan.edu.cn (X.C.)

**Keywords:** gender difference, gender tendency, colorectal cancer, patients, psychosocial outcomes, coping strategies

## Abstract

(1) Background: Gender is an important factor impacting cancer experience. This review mainly aimed to summarize colorectal cancer (CRC) patients’ gender differences in psychosocial outcomes and coping strategies. (2) Methods: Relevant studies were searched for in four electronic databases from 2007 to July 2023. And manual searching was performed on the included studies’ reference lists to identify additional eligible studies. (3) Results: A total of 37 eligible articles were included in this review. These studies were conducted in 19 countries, and they targeted CRC patients at various treatment stages. Significant results showed that female patients tended to have more psychological distress, complex social functioning, and less sexual distress and to choose more positive coping strategies than male patients. But there was no gender difference in psychosocial outcomes and/or coping strategies in some studies, which implied that gender similarity also existed. (4) Conclusions: The findings support the fact that there are both gender differences and similarities in CRC patients’ psychosocial outcomes and coping strategies. A perspective beyond the simple masculine–feminine binary improved our in-depth understanding of gender tendency. Importantly, taking gender tendency into account is critical for medical staff to provide more personalized support and communication interventions.

## 1. Introduction

The GLOBOCAN 2020 data reported that both the incidence and mortality of colorectal cancer (CRC) ranked among the top three of all types of cancer worldwide. Specifically, new CRC cases were the third most common in males and the second most common in females [1]. And CRC incidence is still rapidly growing in many low-income and middle-income countries [2]. Although the trends of CRC incidence are stable or decreasing in some high-income countries, its actual incidence remains among the highest in the world [2]. Additionally, thanks to early detection and advanced treatment, the 5-year survival rate of CRC patients has reached 65%, which means that there may be more CRC patients who need to be taken care of in the future [3].

As a type of life-threatening and long-term illness, CRC causes psychosocial distress to patients over its whole trajectory. At the time of diagnosis and in the following several years, CRC patients present a growing risk of anxiety and depression [4], with the average prevalence ranging from 1.0 to 47.2% and from 1.6 to 57%, respectively [5]. Surgery, chemotherapy, and radiotherapy are the main treatment methods for CRC patients. Despite recent advances, the treatment of CRC and its subsequent side effects still inevitably cause physical symptoms in patients, like fatigue, abdominal pain, diarrhea, constipation, nausea, vomiting, and/or peripheral neuropathy [6]. These physical symptoms can further trigger shock, distress, and emotional unpreparedness in patients [7]. During follow-up appointments, CRC patients generally feel anxiety and fear of recurrence/progression, especially evident in patients with advanced cancer, which may originate from the fear of experiencing adverse side effects again from additional treatment [7]. Even for cancer-free CRC patients, they are also likely to suffer from some treatment-related side effects and long-term psychological distress [8]. In addition, body image distress is another most common CRC-related psychological symptom [6], which primarily stems from surgical scars, weight loss, and stoma bags [7]. Specifically, patients with a stoma were found to perceive worse and persistent body image distress than those without a stoma [9].

CRC also negatively influences patients’ social functioning (e.g., relationships with others). Common physical symptoms, like bowel dysfunction, stoma-related problems (e.g., leaks, blockages, and gas), and altered appearance, were found to evoke shame in CRC patients and concerns about contact with others, which made them unconfident to take part in social activities and feel further isolated from the social environment [7,9]. In addition, preoperative radiotherapy, complications during or after surgery, and a stoma were proven to be accurate predictors of increased sexual dysfunction [10]. Furthermore, sexual dysfunction, stoma-induced embarrassment, and self-consciousness could increase CRC patients’ sexual distress and then undermine their romantic relationships to some extent [7,10]. Some experimental studies also pointed out that general anxiety and depression, body image distress, and sexual distress were negatively associated with CRC patients’ social functioning [11,12].

To face the adversity, CRC patients choose a series of coping strategies, such as positive reappraisal, seeking or receiving social support, and acceptance of the fact [7]. A review summarized that improvements in coping capability positively impacted CRC patients’ emotions and quality of life (QOL) [13]. However, some CRC patients refused social support from others or did not want to talk about anything related to cancer, which may be a result of their negative stigma or some self-perceived burden [14,15]. Brennan provided a reasonable explanation in the Social–Cognitive Transition (SCT) model of adjustment; that is, adjustment to cancer is a psychological process, and time and transition are required to adapt to cancer gradually [16]. For example, avoidance may be attributed to the difficultly in accepting destructive information at that moment, which is a temporary form of self-protection [16]. What we should value is the identification of patients’ maladaptive coping strategies during the adjustment process and give them some supportive interventions.

Importantly, literature reviews mentioning the impact of gender on CRC patients’ psychological outcomes similarly stated that female patients experienced more anxiety, depression, psychological distress [4], and body image distress [6,9], which is in accordance with a systematic review focusing on gender differences in cancer patients’ experiences [17]. The systematic review also analyzed gender differences in coping, but no consistent conclusion was made due to conceptual differences, varied measurement tools, and limited studies [17]. Grant et al. explored gender differences in several domains of QOL of CRC patients with a qualitative approach [18]. They found that the issues of depression and body image were only discussed by female patients, while male patients disclosed little or no difficulties in adapting to cancer. According to these studies [4,6,9,17,18], female patients seemed to be a subgroup more vulnerable to CRC. However, to make more rigorous conclusions, it is necessary to systematically review the studies emerging in recent years that involved gender-related analyses about CRC patients’ psychosocial outcomes and/or coping strategies. An evidence-based exploration and in-depth understanding about the impact of gender on CRC patients’ adjustment are critical to guide future specific interventions.

In particular, in order to better define the scope of focus of this review, it is necessary to clarify the concepts of the terms “sex” and “gender” and how we treat them in this review. Currently, in humans, it is widely recognized that sex refers to a set of biological attributes, like chromosomes, hormone function, and reproductive/sexual anatomy [19]. In contrast, gender refers to the socially constructed roles, behaviors, and identities of women, men, and gender-diverse people [19]. All of the included studies (that is, the studies in Table 1) conducted gender-related analyses based on comparisons between physiological females and males, so, in line with this, the terms females and males in this review refer to physiological and binary sex. But our focus was female and male patients’ psychosocial responses to CRC and their coping behaviors rather than their physiological index (e.g., the pharmacokinetics and pharmacodynamics of pharmaceutical agents). In addition, we attempted to explain CRC patients’ significant gender differences in psychosocial outcomes and coping strategies from a social–culture perspective. As a result, it was considered appropriate to use gender as the collective term for females and males in this review.

### Data Extraction and Synthesis

A table was constructed to extract and synthesize the included studies’ critical information. The content included author, publication year, the country of study, study design, the number of participants, the time points or intervals of study, measurement tools, and main conclusions. Detailed information is shown in Table 1.

In sum, this systematic review aimed to (a) summarize CRC patients’ gender differences in psychosocial outcomes and coping strategies and to (b) provide some potential advice for future research after exploring in-depth the reasons behind the gender differences. Notably, given that a similar review was conducted based on a literature search up to May 2007 [17], this review only included eligible studies published after this time point.

## 2. Materials and Methods

### 2.1. Search Strategy

**Figure 1:**
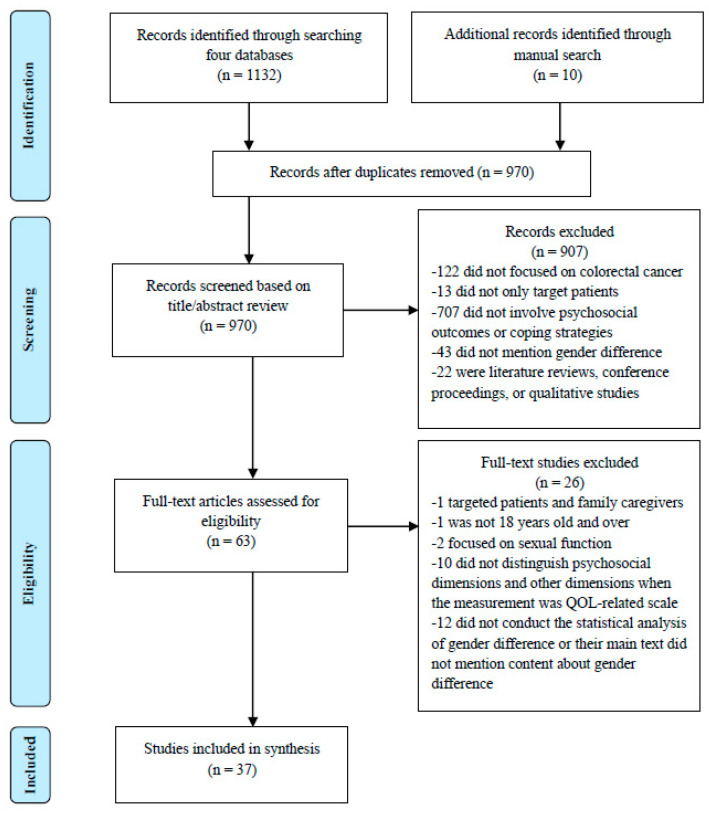
A flow diagram depicting how the relevant literature was identified.

This review was performed under the guidelines of PRISMA (Preferred Reporting Items for Systematic Reviews and Meta-Analyses). A systematic search was conducted to identify eligible quantitative studies in four electronic databases (CINAHL, Embase, PsycINFO, and PubMed) from 2007 to July 2023. The key terms used in the title and/or abstract screening were as follows: (“colorectal cancer” or “colorectal tumor” or “colorectal oncology” or “colon cancer” or “rectal cancer”) AND (“patients” or “survivor”) AND (“gender” or “sociodemographic characteristic” or “sociodemographic factors” or “women and men” or “female and male”) AND (“psychology” or “distress” or “anxiety” or “depression” or “benefit finding” or “relationship satisfaction” or “quality of life” or “coping” or “adjustment”). The search results were limited as follows: articles were written in English or Chinese, and patients’ age was 18 or older. A manual search was also performed on the included studies’ reference lists to pick out additional eligible studies. Figure 1 shows a flow diagram depicting how the relevant literature was identified.

### 2.2. Selection Criteria

The inclusion criteria were as follows: the study (a) targeted CRC patients, whose age was 18 or over; (b) focused on cancer-related psychosocial outcomes and/or coping strategies; (c) conducted a statistical analysis of gender differences; and (d) was a complete report in English or Chinese in peer-reviewed journals.

The exclusion criteria were as follows: the study (a) targeted informal caregivers or couples; (b) did not distinguish psychosocial dimensions and other dimensions when the measurement was a QOL-related scale; (c) main text did not mention content about gender difference; and (d) was a conference proceeding, review, qualitative article, or study protocol.

### 2.3. Quality Assessment

Given that all of the included studies were cross-sectional or longitudinal articles, we chose to utilize the JBI Critical Appraisal Checklist for Analytical Cross-Sectional Studies to assess the included studies’ quality [55]. Specifically, this assessment tool [56] classifies studies as having a “low” (0~2 “yes”), “moderate” (3~4 “yes”), or “high” (>5 “yes”) methodological quality based on eight questions. Table S1 shows the detailed evaluation standard and quality ranking.

To ensure the accuracy and rigor of this review, two trained reviewers independently screened the retrieved studies, extracted and synthesized the included studies’ information, and assessed their quality ranking. Discrepancies were first resolved by the two reviewers. With respect to the discrepancies that were difficult to resolve, a team group discussion was conducted to reach a consensus opinion.

## 3. Results

### 3.1. Study Selection Process

A total of 1142 studies were found in four electronic databases and through additional manual searching. Firstly, 172 duplicates were deleted with the assistance of Endnote 20. Secondly, of the remaining 970 studies, 907 were excluded after screening their titles and abstracts, and then 63 full-text studies were reviewed according to the inclusion and exclusion criteria. Finally, we identified 37 eligible studies for the final analysis (see Figure 1). The primary reasons for excluding studies were that they did not focus on CRC or did not involve psychosocial outcomes or coping strategies.

### 3.2. Quality of the Included Studies

Of the included 37 studies, 25 studies were classified as high quality, and the remaining 12 were classified as moderate quality (see Table S1). The reasons of negatively impacting studies’ quality were mainly an inadequate description of the inclusion criteria (e.g., unclear survey time points) and not controlling for confounders. We finally decided to include all of the 37 studies because they involved a gender-related statistical analysis of psychosocial outcomes and/or coping strategies, and their consistent or contradictory results deserved our deep consideration and exploration.

### 3.3. Characteristics of the Included Studies

The included 37 studies had been conducted in 19 countries from 2008 to 2022, of which the top 3 were the USA (n = 10; 27%), China (n = 4; 10.8%), and Iran (n = 3; 8%). Of the 37 studies, 6 were multicenter cross-sectional studies, 20 were single-center cross-sectional studies, 6 were multicenter longitudinal studies, and 5 were single-center longitudinal studies. These studies started surveys at different time points, covering 3 months to 16 years after diagnosis. And the follow-up periods of 11 longitudinal studies ranged from 1 month to 5 years. The total number of participants in the included studies was 17,141, varying from 40 to 3734. Of the 37 studies, 1 targeted patients aged 60 or above [36], 1 targeted patients whose age was 80 or over [33], and the remaining 35 targeted patients aged from 19 to 88. Furthermore, 7 studies focused on patients with ostomy [27,30,34,40,44,45,46], and the remaining 30 focused on patients with or without ostomy, or they did not mention whether the patients had an ostomy. The topics examined with a statistical analysis in terms of gender differences were divided into three aspects: psychological outcomes, social outcomes, and coping strategies. Psychological outcomes included anxiety, depression, body image distress, distress, fear of cancer recurrence, emotional functioning, psychological functioning, mental health, emotional well-being, spiritual well-being, psychological health, and benefit finding. Social outcomes included sexual distress, sexual satisfaction, relationship satisfaction, and social functioning of QOL. Coping strategies comprised adjustment, distress disclosure, coping capability, resourcefulness, spirituality, and social support. These indicators were measured using various tools. Detailed information about the characteristics of the included studies is presented in Table 1.

### 3.4. Gender Differences in Psychological Outcomes

There were 32 studies exploring gender differences in patients’ psychological outcomes (detailed content shown in Table 1). In general, female patients reported worse anxiety (at least 6 months of survival left) [11,21,23], depression (at least 1 month after treatment, or within 3.5 to 5 years after diagnosis) [21,29,41], body image distress (within 5 years after diagnosis, undergoing chemotherapy treatment, after treatment, or at least 6 months of survival left) [12,20,23,40,51], distress (at 3 months after treatment) [32], fear of cancer recurrence (within 6 to 36 months after treatment) [43], emotional functioning (at least 3 months of survival left) [35], psychological functioning [44], and mental health (within 1 month after diagnosis) [47] than male patients. This gender tendency of anxiety, depression, and distress remained at follow-up periods from 6 months to 55 months [31,49,53]. Considering the moderators, Baldwin et al. reported that, for patients with less sleep disruption or high fatigue, females had worse mental health than males (at least 5 years of survival left) [27]. Belachew and colleagues found that, in white patients only, females perceived lower mental health than males [28].

However, three studies revealed the opposite result that male patients were more likely to have depression [11] and distress [31,36] than female patients. Goldzweig and colleagues explained that male patients may have a worse ability to acquire social support [36]. Dunn et al. speculated that male patients were less likely to seek health services and that young, poor males needed specific support [31], while Mols et al. did not discuss this point [11]. In addition, there were also 19 studies stating that no gender difference existed in anxiety, depression, body image distress, distress, emotional functioning/well-being, psychological health, or spiritual well-being [12,20,21,22,24,25,29,37,38,39,40,41,42,45,46,49,50,51,52]. Specifically, of the 19 studies, 14 utilized QOL-related tools to measure patients’ psychological outcomes.

A longitudinal study focusing on the benefit finding of CRC patients was also included, in which female patients perceived higher benefit finding than male patients at baseline, but as benefit finding increased, this gender difference disappeared by the 6-month follow-up [54].

### 3.5. Gender Differences in Social Outcomes

A total of 16 studies analyzed the gender differences in patients’ social outcomes. As shown in Table 1, the results on gender differences in social functioning are complex. Three articles demonstrated that female patients had worse social functioning than male patients (regardless of stage or treatment) [21,22,39], which may be due to the fact that male patients attempted not to show their weaknesses or dependence [22] or that female patients had a lower tolerance threshold against stressors [39]. Conversely, two other studies reported that female patients showed better social functioning than male patients (at 9 months after diagnosis or at 12 months after surgery) [38,52], even at the 10-month follow-up [52]. Kinoshita and colleagues thought that this may be due to the longer of duration treatment in male patients [38], while Yost et al. attributed it to the different measurements used [52]. In addition, eight studies did not find a significant difference in social functioning/well-being between female and male patients [20,24,35,40,42,45,48,51].

With regard to sexual relationships, three studies stated that no gender difference existed in sexual distress or marriage/relationship satisfaction [12,30,41]. One study reported that female patients had a higher sexual satisfaction than male patients [21], but the original study did not clearly explain this finding.

### 3.6. Gender Differences in Coping Strategies

There were six studies analyzing gender differences in patients’ coping strategies (detailed information shown in Table 1). Generally, female patients seemed to adopt more positive coping strategies than male patients. For example, female patients had better self-disclosure (after colostomy) [30] and fighting spirit to cope with CRC, while male patients felt helpless and turned to fatalism for help (within 2 to 6 years after diagnosis) [36]. Gautam and colleagues also reported that female patients showed more acceptance of CRC and took part in more social engagements (at least 6 months after colostomy) [34]. Consistent with this, Au et al. found that female patients possessed more learned behavior skills, which included personal and social resourcefulness to cope with CRC (after surgery) [26]. In addition, no significant gender difference was found in three studies, including in spirituality (a coping strategy used to manifest the power of life through connections with self, others, the environment, nature, or a higher being that helps people to go beyond themselves and to live life fully) [26], adaptation level (worry, accept, and positive attitude toward life) [30], and coping ability [33]. These seemingly inconsistent results require further consideration and exploration.

As for social support, Eriksen and colleagues targeted patients aged 80 or over, and they showed that females received more affectionate support than males (at least 1 year after surgery) [33]. Goldzweig et al. found that female patients received more support from friends, while male patients received more support from their spouse; all of the patients were 60 years old or over (within 2 to 6 years after diagnosis) [36]. They thought that this gender difference was due to the cultural mores of Israeli society, in which males are expected to become a “hero” and reject friends’ support [36]. Another study found no gender difference in social support between female and male patients [41].

## 4. Discussion

This review summarized CRC patients’ gender differences in psychological outcomes, social outcomes, and coping strategies from a quantitative viewpoint. According to the significant results, female patients tended to have more psychological distress/worse psychological functioning, complex social functioning, and less sexual distress and to choose more positive coping strategies than male patients. And this gender impact was displayed over a wide range of the CRC trajectory. It should also be noted that there were some inconsistent results, which challenged the simple binary conclusion about gender impact, that is, female patients seemed to be a subgroup more vulnerable to CRC. Herein, we attempted to consider the underlying reasons behind the phenomenon of gender differences and to provide some potential advice for future research.

### 4.1. Potential Explanation of Gender Differences in Psychological Outcomes

When knowing the diagnosis of CRC, both female and male patients feel somewhat shocked [57]. It was found that females were more willing to express their emotional response through crying or emotional talk, while males had “unemotional” reactions [57]. In addition, CRC patients generally experience long-term side effects after treatment, with females being more comfortable to admit a sense of feeling in “limbo” due to persistent side effects and most males recounting no problems in this regard [58]. These representations provide a potential reason for why female patients report more psychological distress. Female and male patients might experience similar cancer-related stresses, but females tend to express them to help detection, while males refuse to expose their own weakness publicly [58]. The deep-rooted reasons could stem from socially constructed traditional gender roles; that is, females are characterized as being expressive [59], and males are expected to be stoical [60]. With regard to body image distress, its prevalence had a higher tendency in recent years, ranging from 25.2% to 80.1% [9]. This review shows that female gender was highly proved as a risk factor for body image distress, perhaps because females attach more importance to their appearance and consider it as their emotional strength [61].

As for the male patients’ proneness to depression and distress, the original authors attributed these to the performance of vulnerable subgroups (e.g., young and poor male patients) or the lack of seeking social support [31,36]. McCaughan et al. specifically stated that male patients felt angry about cancer-induced difficulties and humiliated for passively receiving their wife’s care in stoma management [57], because receiving other’s care/support threatened their independence and their identity as a protector [60]. On the contrary, female patients actively sought and accepted emotional support from various sources [62], which played a critical role in reducing their distress. Compared with female patients, some male patients could perceive additional ambivalent distress; that is, they could be caught in a dilemma between needing help and being ashamed to seek support due to ideal hegemonic masculinity [60]. Moreover, Shi and colleagues proposed that females’ depression was mostly identified as mild/moderate, while males’ depression was mostly identified as severe, which is also why males had a higher prevalence of suicide [63]. This finding alerted us to pay more attention to male patients, particularly when they reported higher rates of depression.

### 4.2. Potential Explanation of Gender Differences in Social Outcomes

Limited studies in this review exhibited an almost opposite gender tendency of social functioning, which may indicate that both female and male patients suffer from social dysfunction; nevertheless, their compensation strategies for that are different. For example, it was found that, when it came to outdoor entertainment activities, male patients with a stoma were inclined to adjust their diet in advance, become familiarized with the location of public toilets, or carry an emergency box in order to ensure successful outing, while female patients with a stoma tended to decrease their social activities [57]. On the contrary, female patients talked more about cancer-related concerns with others [64], which naturally promoted their emotional interactions with surrounding people. And self-disclosure was also beneficial for patients’ relationship with their partner [30]. One study demonstrated that female patients had higher sexual satisfaction, which may be a result of them having fewer sexual problems or lower sexual interest than male patients [21,50,51,65,66].

### 4.3. Potential Explanation of Gender Differences on Coping Strategies

Aligned with this review, Tamres and colleagues concluded that females preferred using more kinds of coping strategies to deal with various stressors, regardless of whether they were emotion-focused coping strategies (e.g., active coping and seeking instrumental support) or problem-focused coping strategies (e.g., avoidance, positive reappraisal, and seeking emotional support) [67]. One potential assumption was that female patients engaged in more coping strategies in an effort to achieve inner balance, as they appraised the stressors as more severe and distressed than male patients [67]. But this conjecture requires more research for verification.

One interesting finding was that female patients received more support from friends, while male patients received more support from their spouses [36]. Studies consistently found that female patients tended to seek emotional support from their friends, their families, and clinical nurses [62], but they complained about their husband’s inability to share their feelings [68]. In contrast, male patients commonly only expressed their feelings to spouses in private, who they had established a strong relationship with [57,62]. Both the former and the latter reflect cancer patients’ need to share their emotional responses with intimate spouses, though the situations are different. Indeed, studies evidenced that mutual constructive communication within cancer couples was positively associated with patients’ and spouses’ psychosocial outcomes [69,70]. A systematic review concluded that couple-based communication interventions could improve cancer patients’ and/or their spouses’ relationship functioning (e.g., intimacy and relationship satisfaction) and individual functioning (e.g., anxiety and depression), particularly for those with distress or communication difficulties before intervention [71]. However, when it comes to the enlightenment of gender differences for future communication interventions, we firstly need to acknowledge the fact that there is an overlap between gender performances, like stressor appraisal, emotional responses, and coping strategies [57,58,68], which is why some studies in this review did not find gender differences between psychosocial outcomes and coping strategies. Under the premise of meeting common needs, we could focus the conversation on emotional issues for female patients and instrumental issues for male patients. Sometimes, information support was also considered as a type of emotional support by male patients [62]. Additionally, we could attempt to guide patients to re-affirm/reformulate their ideas of masculinity (or femininity) [72,73], like reformulating help seeking to be seen as an action solving a problem and responding to their wife’s emotions to be seen as an action protecting her mental health, which are considered appropriate masculine acts.

It needs to be noted that mutual communication within cancer couples is an interactive process involving the two roles of patient and spouse. Simultaneously considering the gender tendency of spouses would be helpful in designing more effective communication interventions. Aylaz and colleagues reported that, compared with female spouses, male spouses spent less time with their wife when she was diagnosed with CRC from pre-operation to 18 months post-operation [74]. Regarding female patient–male spouse couples, male spouses tended to initiate discussion about cancer-related issues to help their wife solve problems but withdraw in response to their wife’s emotional expression [75]. In contrast, previous studies revealed that female patients had a greater desire to receive ongoing support to cope with cancer-related physical and psychological difficulties [58] and needed more emotional support than male patients [57,62]. Such findings suggest that male spouses should pay long-term attention to their wife’s needs and make more effort to listen to and respond to their wife’s emotional disclosure. Additionally, two studies reported that the female spouses of CRC patients perceived more and longer-term psychological distress than male spouses [76,77]. Another study targeting the spouses of CRC patients showed that only for female spouses was social support (e.g., spousal support) positively related to their finding meaning [78]. As for male patient–female spouse couples, most male patients were reluctant to express their emotional responses, while female spouses wanted more emotional reactions and information from their husbands [75]. Sometimes, the avoidance of talking about cancer-related emotions is a form of protection for both parties, but appropriate expression at the right time is beneficial for couples’ emotional interaction and distress release [79]. Such evidence suggests that male patients should express their thoughts and feelings more, at least when their wives really want to know.

### 4.4. Clinical Implications

Exploration of the gender tendency of CRC patients’ psychosocial outcomes and coping strategies provides medical staff with a macro-context, which can help them notice CRC patients’ preferences and similarities when offering support. Additionally, discussion about the gender tendency of spouses’ cancer experience helps medical staff to know how to specifically guide mutual communication within cancer couples, which is the key process of promoting dyadic coping with and adaptation to cancer [80]. However, it should be emphasized that CRC patients, regardless of gender, may show similar stress reactions and have common unmet needs when they are faced with this life-threatening disease. The gender tendency of psychosocial outcomes and coping strategies is actually a kind of distinctive manifestation of stress reactions. To neglect the common unmet needs of CRC patients and only focus on gender tendency is putting the cart before the horse. It is not suggested that medical staff treat CRC patients a priori from a gender perspective. The recommendation of this review is that, when CRC patients perform gender-specific reactions and these reactions hinder their effective coping with cancer, medical staff should understand the reasons and, as aforementioned, meet their specific needs and guide them to re-affirm/reformulate their ideas of masculinity (or femininity). In sum, it is important and meaningful to recognize gender tendency in order to provide specific support, particularly for couples coping with cancer, as gender is a key factor embedded in marital relationships.

### 4.5. Limitations of this Review

This review has three primary limitations. Firstly, most of the included studies assessed gender differences as a secondary analysis rather than the main analysis, which impaired the exploration of the impact of gender. But the benefit was to help avoid the “file-drawer” problem wherein studies with significant results are more likely to be published. Secondly, the use of different measurement tools for the same indictor caused difficulties in drawing a conclusion. For instance, most studies reporting significant gender differences in psychological outcomes utilized non-QOL-related scales, while the majority of studies showing no gender differences in psychological outcomes used QOL-related scales. Maybe the psychological outcomes measured via QOL-related scales lack sensitivity for the analysis of gender differences. Thirdly, different countries, or different regions of a country, may treat gender issues differently, which could make patients express their gender identity by simply stating their physiological sex in accordance with social–cultural expectations due to the fear of revealing “otherness”, social desirability, or religious concerns. This query warrants more research.

## 5. Conclusions

This review achieved the aims of systematically summarizing CRC patients’ gender differences in psychosocial outcomes and coping strategies from emerging studies and providing some potential advice for future research after exploring in-depth the reasons behind the phenomenon of gender differences. One crucial finding is that gender differences go beyond the simple masculine–feminine binary. A systematical review published in 2009, which focused on the gender differences in experienced outcomes of patients with CRC and other types of cancer, also seems to be in line with this finding [17]. But more research is needed to determine whether the recommendations proposed in this review are applicable to other types of cancer. To sum up, when medical staff provide support to CRC patients or communication interventions to CRC couples, they should take notice of the gender tendency of patients’ and spouses’ cancer experience, understand their non-traditional masculine or feminine acts, and then improve the effectiveness of the support and interventions.

## Figures and Tables

**Table 1:** CRC patients’ experience in psychosocial outcomes and coping strategies.

Author (Year) Country [Reference]	SD	No. of Participants (Distribution of Gender); Time Points/Intervals	Measurement Tools	Main Conclusions
Acevedo-Ibarra et al. (2021) Mexico [20]	C; Single-center	192 CRC patients (90 women, 102 men); **T**: after diagnosis	-European Organization for Research and Treatment of Cancer Quality of Life Questionnaire (EORTC QLQ)-C30 -EORTC QLQ-CR29 -Impact of Event Scale (IES)	**-Psychological outcomes** • Female patients had worse body image (77.2 vs. 86.6, *p* < 0.001) than male patients; ∗ No gender difference existed in emotional functioning and anxiety. -Social outcomes ∗ No gender difference existed in social functioning.
Akyol et al. (2015) Turkey [21]	C; Single-center	105 colon cancer patients (33 women, 72 men); **T**: unclear	-Hospital Anxiety and Depression Scale (HADS) -EORTC QLQ-C30 -Golombok–Rust Inventory of Sexual Satisfaction (GRISS)	**-Psychological outcomes** • Female patients had more anxiety (9.88 vs. 6.00, *p* < 0.001) and depression (8.03 vs. 5.28, *p* = 0.018) than male patients; ∗ No gender difference existed in emotional functioning. **-Social outcomes** Female patients reported worse social (53.03 vs. 74.76, *p* = 0.007) functioning than male patients; Female patients had higher sexual satisfaction than male patients (4.77 vs. 3.81, *p* = 0.02).
Akyol et al. (2015) Turkey [22]	C; Single-center	222 CRC patients (80 women, 142 men); **T**: unclear	-EORTC QLQ-C30	**-Psychological outcomes** ∗ No gender difference existed in emotional functioning. **-Social outcomes** Female patients had worse social functioning (62.39 vs. 72.67, *p* = 0.026) than male patients.
Al-Shandudi et al. (2022) Oman [23]	C; Multicenter	118 CRC patients (59 women, 59 men); **T**: at least 6 months of survival left	-EORTC QLQ-CR 29	**-Psychological outcomes** Female patients had worse body image (β = 10.7, *p* = 0.038) and anxiety (β = 16.5, *p* = 0.013) than male patients.
Alabbas et al. (2016) Saudi Arabia [24]	C; Single-center	40 CRC patients (15 women, 25 men); **T**: unclear	-FACT-C	**-Psychological outcomes** ∗ No gender difference existed in emotional well-being. **-Social outcomes** ∗ No gender difference existed in social life.
Aminisani et al. (2021) Iran [25]	C; Single-center	303 CRC patients (136 women, 167 men); **T**: regardless of stage or treatment	-HADS	**-Psychological outcomes** No gender difference existed in anxiety and depression.
Au et al. (2012) China [26]	C; Single-center	120 rectal cancer patients (32 women, 88 men); **T**: after surgery	-Resourcefulness Scale (RS); -Body-Mind-Spirit Well-Being Inventory-Spirituality (BMSWBI-Spirituality) Scale	**-Coping strategies** • Female patients had greater resourcefulness than male patients ($\overset{-}{X}$ = 94.33, SD = 20.64); ∗ No gender difference existed in spirituality.
Baldwin et al. (2009) USA [27]	C; Multicenter	286 CRC patients (118 women, 168 men); **T**: at least 5 years of survival left	-The Short Form-36 (SF-36)	**-Psychological outcomes** For patients with less sleep disruption, females had worse mental composite scale (MCS) results than males (48.2 vs. 54.1, *p* < 0.05); For patients with high fatigue, females had worse MCS results than males (45.1 vs. 49.7, *p* < 0.05).
Belachew et al. (2020) USA [28]	C; Single-center	1132 CRC patients (492 women, 640 men); **T**: unclear	-SF-12	**-Psychological outcomes** In white patients, but not in Hispanic or Black patients, female patients had worse MCS scores than male patients (46.1 vs. 48.9, *p* = 0.0002).
Braamse et al. (2016) The Netherlands [29]	C; Multicenter	91 CRC patients (37 women, 54 men); **T**: 3.5 to 5 years after diagnosis	-Beck Anxiety Inventory (BAI); -Inventory of Depressive Symptomatology (IDS)	**-Psychological outcomes** ∗ No gender difference existed in anxiety; • Female patients were more likely to have depression than male patients (F = 4.51, *p* = 0.04).
Du et al. (2021) China [30]	C; Single-center	390 CRC patients (159 women, 231 men); **T**: after colostomy	-Marital Adjustment Test (MAT); -Distress Disclosure Index Scale (DDI) -Ostomy adjustment inventory-20 scale (OAI-20)	**-Social outcomes** ∗ No gender difference existed in marriage satisfaction. **-Coping strategies** • Female patients had better self-disclosure than male patients; ∗ No gender difference existed in adaptation level (worry, accept, and positive attitude toward life).
Dunn et al. (2013) Australia [31]	L; Single-center	1703 CRC patients (680 women, 1023 men); **T**: at 5, 12, 24, 36, 48, 60 months after diagnosis	-The Brief Symptom Inventory (BSI)	**-Psychological outcomes** Female patients had lower frequency and percentage of psychological distress (particularly on anxiety) than male patients at all timepoints.
Eddington et al. (2021) USA [32]	C; Single-center	238 CRC patients (98 women, 140 men) **T**: 3 months after treatment	-National Comprehensive Cancer Network (NCCN) Distress Thermometer -Impact Thermometer	**-Psychological outcomes** Female patients had higher distress than male patients (5.0 vs. 3.0, *p* < 0.05).
Eriksen et al. (2022) Norway [33]	C; Single-center	54 CRC patients over 80 years old (37 women, 17 men) **T**: at least 1 year after surgery	-Sense of Coherence Scale (SOC); -Medical Outcomes Study–Social Support Survey (MOS-SSS)	**-Coping strategies** ∗ No gender difference existed in coping capability; • Female patients had more affectionate support (15.0 vs. 11.0, *p* = 0.01) than male patients.
Gautam et al. (2016) Nepal [34]	C; Single-center	122 CRC patients (60 women, 62 men); **T**: at least 6 months after colostomy	-Ostomy Adjustment Inventory-23 (OAI-23)	**-Coping strategies** Female patients reported higher acceptance (β = −3.078, *p* = 0.023) and social engagement (β = −2.501, *p* < 0.001) than male patients.
Giesinger et al. (2009) Austria [35]	C; Multicenter	206 CRC patients (97 women, 109 men); **T**: at least 3 months of survival left	-EORTC QLQ-C30	**-Psychological outcomes** Female patients had worse emotional (F = 9.07, *p* = 0.003) functioning than male patients. **-Social outcomes** ∗ No gender difference existed in social functioning;
Goldzweig et al. (2009) Israel [36]	C; Multicenter	339 CRC patients over 60 years old (153 women, 186 men); **T**: within 2 to 6 years after diagnosis	-BSI -IES -Cancer Perceived Agents of Social Support (CPASS) -Mental Adjustment to Cancer (MAC)	**-Psychological outcomes** Male patients experienced more psychological distress than female patients, including global psychological distress (F = 31.67, *p* < 0.01), intrusion (F = 34.44, *p* < 0.01), and avoidance (F = 31.41, *p* < 0.01). **-Coping strategies** Female patients had higher scores on fighting spirit (F = 6.68, *p* < 0.05), while male patients had higher scores on helplessness (F = 19.83, *p* < 0.01) and fatalism (F = 5.93, *p* < 0.05); Female patients received more support from friends (F = 3.82, *p* = 0.05), while male patients perceived more support from their spouse (F = 3.97, *p* < 0.05) and religious–spiritual sources (F = 6.82, *p* < 0.01).
Han et al. (2020) Germany [37]	L; Multicenter	212 CRC patients (75 women, 137 men); **T**: at 6 and 12 months after surgery	-Cancer and Treatment Distress (CTXD)	**-Psychological outcomes** ∗ No gender difference existed in distress at 6 and 12 months after surgery.
Kinoshita et al. (2015) Japan [38]	L; Multicenter	75 rectal cancer patients (33 women, 42 men); **T**: before surgery and 1, 6, 12 months after surgery	-EORTC QLQ-C30 -EORTC QLQ-CR38	**-Psychological outcomes** ∗ No gender difference existed in emotional functioning or body image at any time point; • Before surgery, female patients’ global quality of life (QOL) was predicted by emotional functioning, and their emotional functioning was predicted by fatigue and future perspectives; male patients’ global QOL was predicted by body image; • At 6 months after surgery, female patients’ global QOL was predicted by emotional functioning, and their emotional functioning was predicted by financial difficulties; male patients’ cognitive functioning was predicted by body image. **-Social outcomes** Female patients had better social functioning than male patients at 12 months after surgery (*p* = 0.03); At 12 months after surgery, female patients’ social functioning was predicted by financial difficulties, body image, and defecation problems; male patients’ social functioning was predicted by body image.
Laghousi et al. (2019) Iran [39]	C; Single-center	303 CRC patients (136 women, 167 men); **T**: regardless of stage or treatment	-EORTC QLQ-C30	**-Psychological outcomes** ∗ No gender difference existed in emotional functioning. **-Social outcomes** Female patients had worse social functioning (b = −9.14, *p* = 0.038) than male patients.
Mahjoubi et al. (2012) Iran [40]	C; Single-center	96 CRC patients (54 women, 42 men); **T**: after surgery	-EORTC QLQ-C30 -EORTC QLQ-CR38	**-Psychological outcomes** ∗ No gender difference existed in emotional functioning; • Female patients had worse body image (52.7 vs. 75.1, *p* < 0.001) than male patients. **-Social outcomes** ∗ No gender difference existed in social functioning;
Milbury et al. (2013) USA [41]	C; Single-center	261 CRC patients (117 women, 144 men); **T**: at least 6 months after surgery or at least 1 month after treatment	-Center for Epidemiological Studies Depression Scale (CES-D) -EORTC QLQ-CR38 -MOS-SSS -Abbreviated Dyadic Adjustment Scale (A-DAS)	**-Psychological outcomes** • Female patients were more likely to have depression than male patients (11.7 vs. 8.4, *p* = 0.003); ∗ No gender difference existed in body image. **-Social outcomes** ∗ No gender difference existed in relationship satisfaction. **-Coping strategies** ∗ No gender difference existed in social support.
Mols et al. (2018) The Netherlands [11]	L; Multicenter	2625 CRC patients (1178 women, 1447 men) **T**: in 2010, 2011, 2012, 2013	-HADS	**-Psychological outcomes** Female patients had more anxiety than male patients (*p* < 0.01), while male patients were more likely to have depression than female patients (*p* = 0.02); The gender–time interaction effect was not explored.
Mrabti et al. (2016) Morocco [42]	L; Multicenter	294 CRC patients (135 women, 159 men); **T**: within 3 months after diagnosis	-EORTC QLQ-C30	**-Psychological outcomes** ∗ No gender difference existed in emotional functioning. **-Social outcomes** ∗ No gender difference existed in social functioning.
Palas et al. (2021) USA [43]	C; Single-center	120 CRC patients (61 women, 59 men); **T**: within 6 to 36 months after treatment	-Fear of Cancer Recurrence Inventory (FCRI)	**-Psychological outcomes** Female gender was related to greater fear of cancer recurrence (*p* < 0.05).
Pereira et al. (2012) Brazil [44]	C; Single-center	60 CRC patients (26 women, 34 men); **T**: unclear	-World Health Organization QOL Instrument (WHOQOL)	**-Psychological outcomes** Female patients had worse psychological functioning (60.42 vs. 73.92, *p* = 0.007) than male patients.
Ran et al. (2016) China [45]	C; Single-center	142 CRC patients (39 women, 103 men); **T**: 1 month after colostomy	-WHOQOL	**-Psychological outcomes** ∗ No gender difference existed in psychological health. **-Social outcomes** ∗ No gender difference existed in social relationships.
Reese et al. (2018) USA [12]	L; Single-center	141 CRC patients (59 women, 82 men); **T**: within 5 years after diagnosis and 6-month follow-up	-CES-D -Body Image Scale (BIS) -The Index of Sexual Satisfaction (ISS) -DAS	**-Psychological outcomes** ∗ No gender difference existed in depression within 5 years after diagnosis and 6-month follow-up; • Female patients suffered from more body image distress (9.62 vs 6.03, *p* = 0.005) than male patients within 5 years after diagnosis. **-Social outcomes** ∗ No gender difference existed in sexual distress within 5 years after diagnosis; ∗ No gender difference existed in relationship satisfaction within 5 years after diagnosis. No gender differences in these variables were explored at 6-month follow-up.
Repić et al. (2016) Serbia [46]	C; Single-center	67 CRC patients (33 women, 34 men) **T**: after colostomy	-Quality of Life Questionnaire for a Patient with an Ostomy (QOL-O)	**-Psychological outcomes** ∗ No gender difference existed in psychological and spiritual well-being.
Reyes et al. (2017) USA [47]	C; Single-center	3734 CRC patients (1566 women, 2168 men); **T**: within 1 year after diagnosis	-SF-12	**-Psychological outcomes** Female patients had worse MCS scores (*p* < 0.001) than male patients.
Ristvedt et al. (2009) USA [48]	L; Single-center	80 rectal cancer patients (35 women, 45 men) **T**: soon after initial treatment and 2–5-year follow-up	-FACT-C	**-Social outcomes** No gender difference existed in social well-being.
Tejada et al. (2017) Spain [49]	L; Multicenter	947 CRC patients (344 women, 603 men) **T**: before surgery and 12-month follow-up	-HADS	**-Psychological outcomes** • Female patients had lower improvement in anxiety than male patients (β = 1.047, *p* < 0.0001); ∗ No gender difference existed in depression.
Thong et al. (2019) Germany [50]	C; Multicenter	1176 CRC patients (477 female, 699 male); **T**: within 5 to 16 years after diagnosis	-EORTC QLQ-CR29	**-Psychological outcomes** ∗ No gender difference existed in body image.
Trinquinato et al. (2017) Brazil [51]	C; Single-center	144 CRC patients (72 women, 72 men); **T**: undergoing chemotherapy treatment	-EORTC QLQ-C30; -EORTC QLQ-CR29	**-Psychological outcomes** • Female patients had worse body image than male patients (76.85 vs. 85.96, *p* = 0.023); ∗ No gender difference existed in emotional functioning and anxiety. **-Social outcomes** ∗ No gender difference existed in social functioning.
Yost et al. (2008) USA [52]	L; Multicenter	568 CRC patients (unclear); **T**: at 9 and 19 months after diagnosis	-FACT-C	**-Psychological outcomes** ∗ No gender difference existed in emotional well-being. **-Social outcomes** Female patients had better social well-being than male patients at 9 and 19 months after diagnosis.
Zhou et al. (2021) China [53]	L; Single-center	302 CRC patients (132 women, 170 men); **T**: baseline (discharged from hospital), 1-, 2-, 3-year follow-up	-HADS	**-Psychological outcomes** Female gender was one of independent factors related to baseline (*p* = 0.001, *p* = 0.005, respectively), 1-year (*p* = 0.001, *p* = 0.003, respectively), 2-years (*p* = 0.005, *p* < 0.001, respectively) and 3-years (*p* < 0.001, *p* = 0.001, respectively) anxiety and depression.
Zimmaro et al. (2021) USA [54]	L; Single-center	133 CRC patients (54 women, 79 men); **T**: baseline and 6-month follow-up	-Benefit Finding Scale	**-Psychological outcomes** • Female patients had higher Benefit Finding Scale scores than male patients at baseline (43 vs. 35, *p* < 0.001); ∗ Benefit Finding Scale scores increased from baseline to 6-month follow-up, but the increase did not differ by gender.

Abbreviations: BSI: Brief Symptom Inventory; C, cross-sectional study; CES-D: Center for Epidemiological Studies Depression Scale; CRC: colorectal cancer; DAS: Dyadic Adjustment Scale; EORTC QLQ: European Organization for Research and Treatment of Cancer Quality of Life Questionnaire; FACT-C: Functional Assessment of Cancer Therapy-Colorectal Scale; HADS: Hospital Anxiety and Depression Scale; IES: Impact of Event Scale; L: longitudinal study; MCS: Mental Composite Scale; MOS-SSS: Medical Outcomes Study-Social Support Survey; OAI: Ostomy Adjustment Inventory; QOL: quality of life; SF-36/12: Short Form-36/12; T, time points/intervals; WHOQOL: World Health Organization QoL Instrument; • presents significant gender difference; ∗ presents no gender difference.

## Data Availability

All data used in this study were completely published online.

## Supplementary Materials

The following supporting information can be downloaded at https://www.mdpi.com/article/10.3390/healthcare11182591/s1, Table S1: Methodological Quality of The Included Cross-Sectional Articles.
