# Supplementary material for: Gender Differences in Psychosocial Outcomes and Coping Strategies of Patients with Colorectal Cancer: A Systematic Review
Source: Healthcare (Basel). 2023 Sep 20;11(18):2591. doi: 10.3390/healthcare11182591 (PMC10530630; doi:10.3390/healthcare11182591)
Supplement: Supplementary file 1 [file healthcare-11-02591-s001.zip › healthcare-2605018-supplementary.pdf]

Table S1: Methodological Quality of The Included Cross-Sectional Articles

Reference	Q1	Q2	Q3	Q4	Q5	Q6	Q7	Q8	QR
Acevedo-Ibarra et al. (2021)	Y	Y	Y	Y	N	N	U	N	M
Akyol et al. (2015)	U	Y	Y	NA	N	N	Y	N	M
Akyol et al. (2015)	N	Y	Y	Y	N	N	Y	Y	M
Al-Shandudi et al. (2022)	Y	Y	Y	Y	Y	Y	Y	Y	H
Alabbas et al. (2016)	U	Y	Y	Y	N	N	Y	N	M
Aminisani et al. (2021)	Y	Y	Y	NA	Y	Y	Y	Y	H
Au et al. (2012)	Y	Y	Y	Y	N	N	Y	Y	H
Baldwin et al. (2009)	Y	Y	Y	Y	N	N	Y	N	M
Belachew et al. (2020)	U	Y	Y	Y	Y	Y	Y	Y	H
Braamse et al. (2016)	Y	Y	Y	Y	Y	Y	Y	Y	H
Du et al. (2021)	Y	Y	Y	Y	Y	Y	Y	Y	H
Dunn et al. (2013)	Y	Y	Y	Y	Y	Y	U	Y	H
Eddington et al. (2021)	Y	Y	U	Y	Y	Y	Y	Y	H
Eriksen et al. (2022)	Y	Y	Y	Y	N	N	Y	Y	H
Gautam et al. (2016)	Y	Y	Y	Y	Y	Y	U	Y	H
Giesinger et al. (2009)	Y	N	Y	Y	N	N	Y	N	M
Goldzweig et al. (2009)	Y	Y	Y	Y	Y	Y	U	Y	H

Han et al. (2020)	Y	Y	Y	Y	Y	Y	Y	Y	H
Kinoshita et al. (2015)	Y	Y	Y	Y	Y	Y	Y	Y	H
Laghousi et al. (2019)	Y	Y	Y	Y	Y	Y	U	Y	H
Mahjoubi et al. (2012)	Y	Y	Y	Y	N	N	U	N	M
Milbury et al. (2013)	Y	Y	Y	Y	Y	Y	Y	Y	H
Mols et al. (2018)	Y	Y	Y	Y	Y	Y	Y	Y	H
Mrabti et al. (2016)	Y	Y	Y	Y	N	N	Y	N	M
Palas et al. (2020)	Y	N	Y	Y	Y	Y	U	Y	H
Pereira et al. (2012)	Y	Y	Y	Y	N	N	U	Y	M
Ran et al. (2016)	Y	U	U	U	Y	Y	U	Y	M
Reese et al. (2018)	U	Y	Y	Y	Y	Y	U	Y	H
Repić et al. (2016)	Y	Y	U	Y	N	N	Y	U	M
Reyes et al. (2017)	Y	Y	Y	Y	Y	Y	U	Y	H
Ristvedt et al. (2009)	Y	Y	Y	Y	Y	Y	Y	Y	H
Tejada et al. (2017)	Y	Y	Y	Y	Y	Y	Y	Y	H
Thong et al. (2019)	Y	Y	Y	Y	Y	Y	U	Y	H
Trinquinato et al. (2017)	Y	Y	Y	Y	N	N	U	N	M
Yost et al. (2008)	Y	Y	Y	Y	Y	Y	Y	Y	H
Zhou et al. (2021)	Y	Y	Y	Y	Y	Y	U	Y	H
Zimmaro et al. (2021)	U	Y	Y	Y	Y	Y	Y	Y	H

All quality ranking of included cross-sectional and longitudinal studies were assessed by JBI Critical Appraisal Checklist for Analytical Cross-Sectional Studies. Y: yes; N: no; U: unclear; NA: not applicable. H: High quality; M: Moderate quality; L: Low quality. Q1: Were the criteria for inclusion in the sample clearly defined? Q2: Were the study subjects and the setting described in detail? Q3: Was the exposure measured in a valid and reliable way? Q4: Were objective, standard criteria used for measurement of the condition? Q5: Were confounding factors identified? Q6: Were strategies to deal with confounding factors stated? Q7: Were the outcomes measured in a valid and reliable way? Q8: Was appropriate statistical analysis used?
